# Implementation of a novel TRIZ-based model to increase the reporting of adverse events in the healthcare center

**DOI:** 10.1038/s41598-024-78661-3

**Published:** 2024-11-06

**Authors:** Jiun-Yih Lee, Pei-Shan Lee, Cheng-Hsien Chiang, Yi-Ping Chen, Chiung-Ju Chen, Yuan-Ming Huang, Jlan-Ren Chiu, Pei-Ching Yang, Chen-An Yeh, Jui-Ting Chang

**Affiliations:** 1 Center for Quality Management, Shin Kong Wu Ho-Su Memorial Hospital, No. 95, Wenchang Rd., Shilin Dist., Taipei City 111, Taipei, Taiwan; 2 Department of Pharmacy, Shin Kong Wu Ho-Su Memorial Hospital, Taipei, Taiwan; 3 Department of Information Technology, Shin Kong Wu Ho-Su Memorial Hospital, Taipei, Taiwan; 4 Department of Pathology and Laboratory, Shin Kong Wu Ho-Su Memorial Hospital, Taipei, Taiwan; 5 Department of Engineering, Shin Kong Wu Ho-Su Memorial Hospital, Taipei, Taiwan; 6 Department of Radiological Diagnosis, Shin Kong Wu Ho-Su Memorial Hospital, Taipei, Taiwan; 7 Nursing Department, Shin Kong Wu Ho-Su Memorial Hospital, Taipei, Taiwan; 8 College of Medicine, Fu-Jen Catholic University, Taipei, Taiwan; 9 Division of Nephrology, Department of Internal Medicine, Shin Kong Wu Ho-Su Memorial Hospital, Taipei, Taiwan

**Keywords:** Adverse events, TRIZ, Incident reporting, Healthcare quality, Patient safety, SERVQUAL, Outcomes research, Health policy

## Abstract

Underreporting of adverse events in healthcare systems is a global concern. This study aims to address the underreporting of adverse events (AE) by implementing a TRIZ-based model to identify and overcome barriers to reporting, thus filling gaps in current reporting practices and improving incident recognition. A TRIZ (Theory of Inventive Problem Solving) approach was adopted, integrating with SERVQUAL methodologies to design interventions. Preintervention and postintervention surveys were conducted to evaluate changes in the recognition of adverse events and barriers to reporting. Statistical analyses were performed to assess the effectiveness of the interventions. Recognition improved and barriers to reporting AEs significantly decreased. Monthly reported cases rose from 33.7 to 50.3 (*p* = 0.000), demonstrating the effectiveness of the TRIZ-based interventions. Implementing a TRIZ-based model significantly improved adverse event reporting by enhancing the recognition of reportable events and overcoming identified barriers. Future research should explore the long-term sustainability of these interventions and their broader applicability in diverse healthcare settings.

**Supplementary Information:**

The online version contains supplementary material available at 10.1038/s41598-024-78661-3.

## Introduction

Adverse events (AEs) are frequently underreported in healthcare centers globally, with most reporting systems capturing only 7–15% of all AEs^1^. This high rate of underreporting suggests that we are failing to meet the fundamental goals of learning from errors and enhancing the healthcare system to improve patient safety^2^. To address this issue, both the World Health Organization (WHO) and the Joint Commission of Taiwan (JCT) have established goals and policies to encourage the reporting of AEs. Consequently, under the Medical Malpractice and Dispute Resolutions law, healthcare organizations in Taiwan are responsible for identifying barriers to incident reporting and implementing interventions to increase AE reports. However, while several studies have identified barriers to incident reporting^2–4^, few have implemented interventions to overcome these barriers^5,6^.

SERVQUAL stands for Service Quality and is a practical tool that companies use to quantitatively assess customer feedback and service quality. It evaluates how well a business meets customer expectations across 10 dimensions, including tangibles, reliability, responsiveness, communication, credibility, security, competency, understanding customers, courtesy, and accessibility (later reduced to 5 dimensions by scholars)^7^. SERVQUAL has been widely applied in healthcare settings^8^ to assess the quality needs of both internal customers and external customers^9^. Assessing the quality requirements of both internal and external customers is crucial because these requirements are directly linked to service efficiency and outcomes. Quality requirements refer to specific needs that must be met to ensure optimal service delivery. For internal customers, such as healthcare staff, these requirements include access to adequate workplace equipment, proper training, and support from management, which will increase the number of incident reports^2^. The TRIZ model has been designed to improve service quality^10^. ‘TRIZ’ is an abbreviation of the Russian phrase ‘teorija rezhenija izobretatelskih zadach’, which means Theory of Inventive Problem Solving. TRIZ is an innovation-based methodology, whereas SERVQUAL is a structured quality methodology. Combining the two methodologies may facilitate the effective evaluation of the quality requirements of internal and external customers and the implementation of creative problem-solving to ensure the highest degree of service quality^11^.

The TRIZ-based resolution of management problems requires the validation of many successful cases^12^. Thus far, the only domain accredited by the International TRIZ Association is Business and Management. The lack of data on successful cases and of TRIZ implementation results in the healthcare sector necessitating further studies. The implementation of TRIZ in the healthcare setting requires the translation of quality parameters into TRIZ parameters^13^; the process of translation warrants the use of a contradiction matrix. Although a contradiction matrix of healthcare service quality has been developed for the translation of SERVQUAL parameters into TRIZ parameters^11,14,15^, the use of this matrix and TRIZ import remain to be validated^14–17^. Most studies in healthcare have focused on the development of TRIZ inventive principle and the use of TRIZ for solution development^11,14,15,18^, but not on the effects of the developed solutions.

Considering the aforementioned knowledge gaps, we explored the effect of TRIZ-based model implementation for improving the recognition of AEs and overcoming the barriers to reporting AEs to increase the number of incident reports.

## Methods

### Study setting

The study was conducted at Shin Kong Wu Ho-Su Memorial Hospital (SKH), an 829-bed medical center in Taiwan. SKH utilizes a web-based online reporting system, facilitating healthcare staff in reporting adverse events (AEs) and systematically recording statistics for management purposes. The Joint Commission of Taiwan (JCT) developed the Taiwan Patient Safety Reporting System (TPR), a comprehensive national reporting and communication platform. The TPR mandates the reporting of 14 specific types of AEs in healthcare settings, requiring detailed information about these incidents to be uploaded onto the platform.

### Study design and TRIZ methodology

**Fig. 1:**
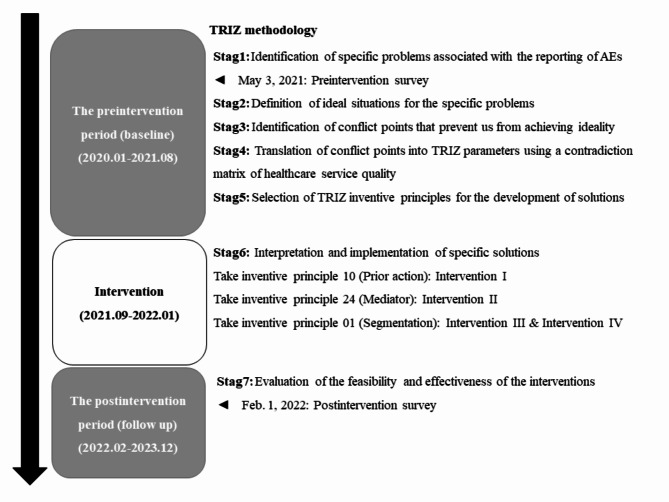
TRIZ stages implemented in the preintervention, intervention, and postintervention periods/*AEs*  Adverse events.

This pre–post study was conducted in three primary periods: preintervention, intervention, and postintervention. The TRIZ methodology was implemented in seven stages (Fig. 1): preintervention, stages 1 to 5; intervention, stage 6; and postintervention, stage 7.

#### The preintervention period was from July 1, 2020, to August 31, 2021

##### Stage 1: Identification of specific problems associated with the reporting of AEs

In this stage, we conducted a preintervention survey to collect data on the barriers to incident reporting. This survey aimed to identify specific obstacles faced by staff, such as time constraints, lack of support, and concerns about repercussions, which could hinder accurate and timely reporting of adverse events. The insights gathered from this survey provided a foundational understanding of the existing challenges, guiding the development of targeted interventions to address these barriers effectively.

##### Stage 2: Definition of ideal situations for the specific problems

After identifying the barriers, we defined the ideal situations as ‘the development of action at the right time without any error or side effect’ and our expectation of improving the specific problems as ‘statistically significant improvement’.

##### Stage 3: Identification of conflict points that prevent us from achieving ideality

We identified the conflict points that prevent ideality from being achieved (Table 1). These points were defined as the negative outcomes associated with the improvement of a specific problem; these outcomes indicate the conflicts between the problems that must be improved and the current system. The identification of conflict points was based on staff interviews and observations of the actual system. In this part of the study, we asked staff to respond based on their past experiences or observations of the system, specifically whether addressing certain barriers could potentially, or had previously, led to other issues (please refer to the supplementary material). The conflict points were defined based on staff feedback collected during this process. For example, one staff member mentioned attempting to speed up the reporting process, which ultimately resulted in incomplete and inaccurate information being submitted.

**Table 1:** Contradiction matrix of healthcare service quality.

Improving parameters (SERVQUAL)	Worsening parameters (TRIZ)	Inventive principles of TRIZ
Access	Energy spent by a moving object	01: Segmentation 13: Doing it in reverse 24: Mediator
Communication	Loss of time	24: Mediator 34: Rejection and regeneration of parts 28: Replacement of mechanical system 32: Change of colour
Understanding	Speed	10: Prior action 28: Replacement of mechanical system 32: Change of colour
Empathy	Speed	35: Translation of properties 10: Prior action 14: Spheroidality
	Loss of time	35: Translation of properties 28: Replacement of mechanical system
Reliability	Complexity of a device	13: Doing it in reverse 35: Translation of properties 01: Segmentation
Tangibles	Amount of substance	36: Phase transition 22: Conversion of harm into benefits
Responsiveness: Time	Reliability	10: Prior action 30: Flexible membranes or thin films 04: Asymmetry
	Accuracy of manufacturing	24: Mediator 26: Copying 28: Replacement of mechanical system 18: Mechanical vibration
Responsiveness: Speed	Accuracy of measurement	28: Replacement of mechanical system 32: Change of colour 01: Segmentation 24: Mediator
	Accuracy of manufacturing	10: Prior action 28: Replacement of mechanical system 32: Change of colour 25: Self-service
Competence	Complexity of a device	02: Extraction 13: Doing it in reverse 25: Self-service 28: Replacement of mechanical system
Assurance	Repairability	01: Segmentation 32: Change of colour 10: Prior action 25: Self-service

##### Stage 4: Translation of conflict points into TRIZ parameters using a contradiction matrix of healthcare service quality

A contradiction matrix of healthcare service quality was developed to translate the aforementioned conflict points into TRIZ parameters. This matrix was developed using the SERVQUAL model^14^, which comprises the following 10 parameters: access, competence, communication, courtesy, credibility, reliability, responsiveness, security, understanding customers, and tangibles(Table 1). For example, reporting speed, related to the SERVQUAL parameter *responsiveness: speed* (improving parameter), may be increased at the expense of information accuracy, related to the TRIZ parameter *accuracy of manufacturing* (worsening parameters). Table 1 summarizes the conflict points.

##### Stage 5: Selection of TRIZ inventive principles for the development of solutions

After the conflict points were translated into TRIZ parameters, appropriate TRIZ inventive principles were selected for the conflict points to develop solutions. For instance, for the conflict between increasing reporting speed (an improving parameter) and decreasing information accuracy (a worsening parameter), the selected TRIZ inventive principles were as follows: 10: prior action, 28: replacement of the mechanical system, 32: change of color, and 25: self-service. These inventive principles were selected through a consensus as the basis for developing four interventions (Table 2). In principle, one appropriate inventive principle is sufficient for overcoming a conflict situation^15^.

**Table 2:** Key stages of the TRIZ methodology.

Stage 1: Identification of specific problems associated with reporting AEs^1^	Stage 3: Identification of conflict points that prevent us from achieving ideality	Stage 4: Use of a contradiction matrix of healthcare service quality to translate conflict points into TRIZ parameters		Stage 5: Selection of appropriate TRIZ inventive principles	Stage 6: Interpretation and implementation of specific solutions
		Improving parameters (SERVQUAL)	Worsening parameters (TRIZ)		
What types of AEs should be reported. Why incident reporting is essential for patient safety.	Increased sensitivity to reporting AEs may result in many nonessential reports, thus increasing the burden on the reporting system.	Responsiveness: Speed	Accuracy of measurement	01: Segmentation	Intervention III
Requirement of a considerable amount of typing information during incident reporting	Increased reporting speed may cause poor information accuracy.	Responsiveness: Speed	Accuracy of manufacturing	10: Prior action	Intervention I
Lack of a reward for incident reporting	Substantial reassurance must be provided to healthcare staff to help them report AEs with confidence; however, the reward system must be revised to ensure an increase in the reporting of only severe AEs and reward staff who actually deserve it.	Assurance	Repairability	01: Segmentation	Intervention IV
Requirement of writing a review report after incident reporting	Although eliminating the requirement of a review report reduces the burden on healthcare staff and increases reporting speed, it may prevent them from learning from their errors.	Responsiveness: Speed	Accuracy of measurement	01: Segmentation	Intervention IV
Time-consuming reporting process	Reduced reporting time may cause poor information reliability.	Responsiveness: Time	Reliability	10: Prior action	Intervention I
Lack of support from supervisors	Supervisors are expected to support staff in reporting AEs; however, some of them may lack leadership skills in the context of ensuring patient safety.	Assurance	Repairability	01: Segmentation	Intervention III
Concerns regarding the effects of incident reporting on colleagues and teamwork	Effective communication is essential; however, the characteristics of different teams vary considerably within a hospital. Moreover, the implementation intervention across the hospital is a time-consuming process.	Communication	Loss of time	24: Mediator	Intervention II
Concerns regarding blame or punishment	Healthcare staff must be reassured that they will not be punished for unintentional errors; this will enable them to report AEs with confidence. However, organisational culture may not be easily changeable.	Assurance	Repairability	01: Segmentation	Intervention IV

^1^AE adverse event.

#### The intervention period started on September 1, 2021

##### Stage 6: Interpretation and implementation of specific solutions

Intervention I: Inventive principle 10 (prior action).

Prior action indicates completely or partially ensuring required changes in an object in advance. Considering this principle, we linked the reporting system to our Healthcare Information System (HIS) and Nursing Information System (NIS) to ensure that the system can automatically complete input data, such as basic patient information and vital signs.

Intervention II: Inventive principle 24 (mediator).

By conducting workshops to educate the staff members assigned by various departments on topics such as patient safety, organizational culture, and teamwork, we developed a long-term partnership with these staff members; they served as mediators for the continual exploration of issues related to patient safety and the promotion of team communication in their departments.

Interventions III and IV: Inventive principle 1 (segmentation).

Segmentation indicates the division of an object into independent parts. Our analysis included the characteristics of different stakeholders, such as supervisory positions and different work environments. For the staff in supervisory positions, we adopted a top-down approach; thus, the superintendent and deputy superintendents of the hospital served as educators who conducted awareness-raising sessions during important staff meetings. We visited different departments to communicate and coordinate with the stakeholders to design customized education programs based on the patient safety incidents and issues encountered in their work environments (Intervention III).

We further designed a reward policy to provide different levels of rewards according to the scores on the severity assessment code (SAC) matrix. Higher levels of AE severity (i.e. lower SAC scores) indicate higher levels of rewards. To reduce the burden of incident reporting, we adjusted the current policy: root cause analysis (RCA) or improvement reports were necessary only for events with SAC scores of 1 or 2 (Intervention IV).

#### The postintervention period started on February 1, 2022

##### Stage 7: Evaluation of the feasibility and effectiveness of the interventions

We conducted a postintervention survey to investigate whether the interventions enabled our healthcare staff to effectively overcome the barriers to reporting AEs and increased the number of incident reports.

### Participants, data collection, and outcome measures

We focused on the following two key outcomes: (1) changes in the recognition of AEs and the barriers to reporting AEs, and (2) changes in the number of incident reports after the interventions. The data collection methods are described below.

Preintervention (May 3 to 14, 2021) and postintervention (February 1 to 14, 2022) surveys were conducted to obtain data regarding the barriers to incident reporting and the recognition of AEs. A questionnaire with a content validity index of 0.92 was used to conduct two anonymous surveys. By performing stratified random sampling using the data provided by the Department of Human Resources in SKH, we collected 423 in preintervention and 203 in postintervention. The recovery rates were 80.3% and 81.4% before and after the interventions, respectively.

The participants’ sociodemographic characteristics, including age, sex, career (years), and designation, were analysed. The recognition of AEs, including the types of AE that should be reported and why incident reporting is essential for patient safety, was assessed through a test. The scores on the test were calculated in terms of percentage values: the number of correct answers divided by the total number of answers. Barriers to incident reporting, such as the requirement of a considerable amount of information during incident reporting, lack of a reward for incident reporting, requirement of writing a review report after incident reporting, time-consuming reporting process, lack of support from supervisors, concerns regarding the effects of incident reporting on colleagues and teamwork, and concerns regarding blame or punishment, were assessed through yes–no questions.

We further recorded the number of incident reports by using data from our Web-based online reporting system. Trends were analysed every months during the study period.

### Statistical analysis

All statistical analyses were performed using SPSS (version 25.0; IBM Corporation, Armonk, NY, USA). The tests were two-tailed, and statistical significance was set at *P* < 0.05.

### Ethics and consent statements

We conducted this study strictly under the Declaration of Helsinki and the requirements of the Institutional Review Board at Shin Kong Wu Ho-Su Memorial Hospital (IRB: 20220502R) and all methods were carried out in accordance with relevant guidelines and regulations. Informed consent was obtained from all subjects or their legal guardians.

## Results and discussion

### Results

**Fig. 2:**
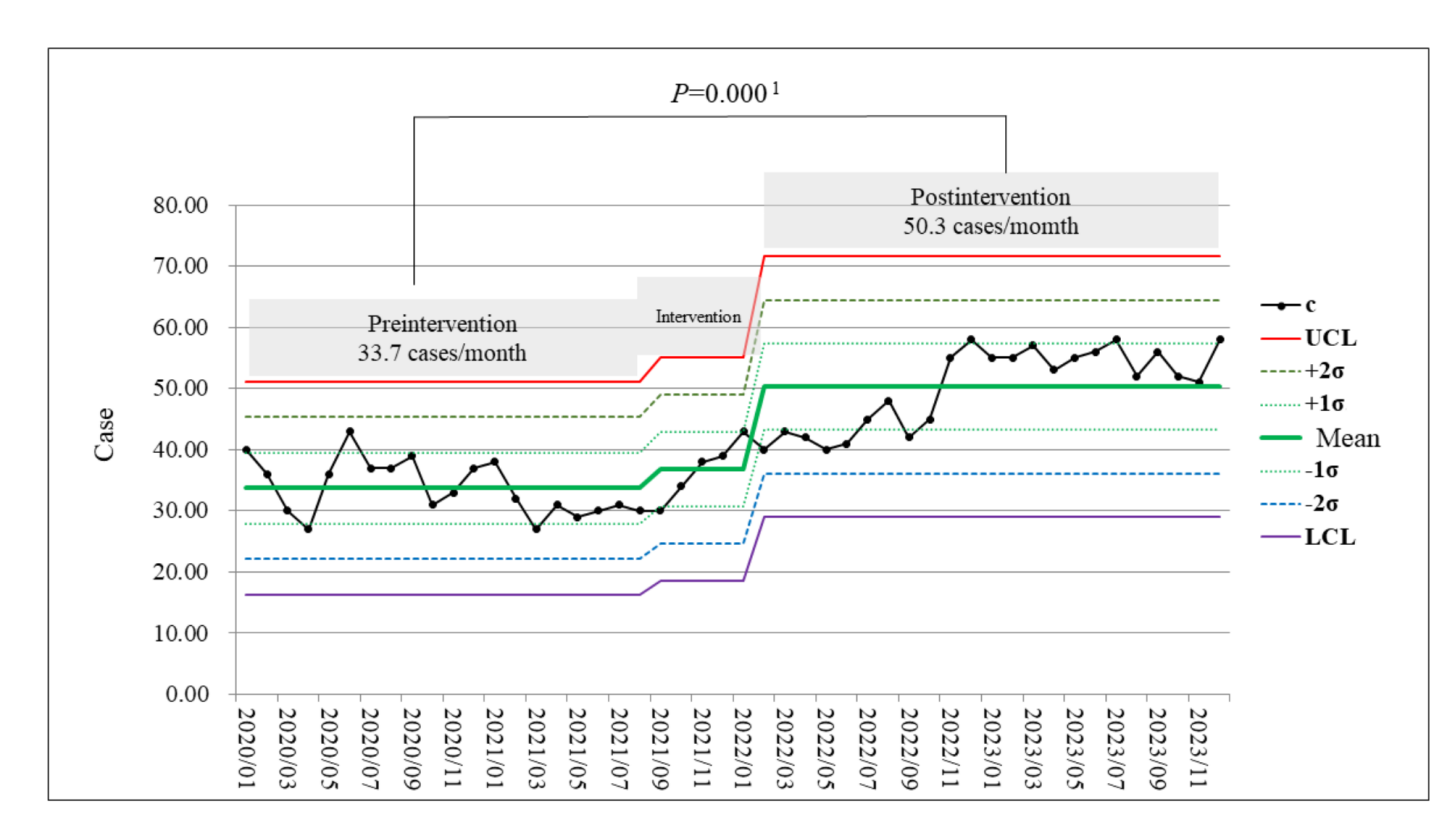
Trends in the reporting of adverse events after the interventions compared with preintervention values/^1^Independent samples t test.

Two anonymous surveys conducted before and after the intervention revealed no significant demographic differences between the preintervention (*N* = 423) and postintervention (*N* = 203) groups. Recognition of AEs improved, with understanding of reportable AEs increasing from 58.6 to 88.4% (*p* < 0.01), and the importance of reporting for patient safety from 75.3 to 85.8% (*p* = 0.000) (Table 3). After the interventions, there were significant reductions in barriers to reporting AEs: The requirement of a considerable amount of information during incident reporting decreased from 56.5 to 23.2% (*p* = 0.000), lack of rewards from 43.7 to 10.3% (*p* = 0.000), the requirement for review reports from 58.6 to 19.7% (*p* = 0.000), the time-consuming process from 75.4 to 29.1% (*p* = 0.000), lack of supervisor support from 50.8 to 22.2% (*p* = 0.000), concerns about the impact on colleagues and teamwork from 46.3 to 20.7% (*p* = 0.000), and concerns about blame or punishment from 52.7 to 40.4% (*p* = 0.040) (Table 3). The mean number of reported cases per month increased from 33.7 to 50.3 (*p* = 0.000) (Fig. 2), demonstrating the success of the TRIZ-based interventions.

**Table 3:** Preintervention and postintervention assessments of the participants’ sociodemographic characteristics, barriers to reporting AEs, and recognition of AEs.

Variables	Preintervention (*N* = 423)	Postintervention (*N* = 203)	*P*
Healthcare staff, n (%)			
Doctor	67(15.8)	28(13.8)	0.916^1^
Nurse	104(24.6)	53(26.1)	
Medical technician	163(38.5)	79(38.9)	
Administrator	89(21.0)	43(21.2)	
Men, n (%)	124(29.3)	73(36.0)	0.094^1^
Career (years), n (%)			
≤ 5	114(27.0)	65(32.0)	0.376^1^
6 to 10	76(18.0)	31(29.0)	
> 10	233(55.1)	107(52.7)	
Nonsupervisory position, n (%)	359(84.9)	163(80.3)	0.169^1^
Age (years), n (%)			
20 to 25	51(12.1)	22(11.7)	0.517^1^
26 to 30	57(13.5)	17(11.8)	
31 to 35	56(13.2)	20(12.1)	
36 to 40	41(9.7)	24(10.4)	
41 to 45	63(14.9)	25(14.1)	
46 to 50	65(15.4)	39(16.6)	
≥ 51	90(21.3)	56(23.3)	
Recognition of AEs, mean (SD)			
What types of AEs should be reported	58.6 ± 27.6	88.4 ± 19.3	0.000^2^
Why incident reporting is crucial for patient safety	75.3 ± 21.9	85.8 ± 18.5	0.000^2^
Barriers to reporting AEs, n (%)			
Requirement of a considerable amount of information during incident reporting	239(56.5)	47(23.2)	0.000^1^
Lack of a reward for incident reporting	185(43.7)	21(10.3)	0.000^1^
Requirement of writing a review report after incident reporting	248(58.6)	40(19.7)	0.000^1^
Time-consuming reporting process	319(75.4)	59(29.1)	0.000^1^
Lack of support from supervisors	215(50.8)	45(22.2)	0.000^1^
Concerns regarding the effects of incident reporting on colleagues and teamwork	196(46.3)	42(20.7)	0.000^1^
Concerns regarding blame or punishment	223(52.7)	82(40.4)	0.004^1^

^1^Chi-square test.

^2^ Independent samples *t* test.

*AE* adverse event.

## Discussion

### Principal findings

The TRIZ-based model significantly enabled our healthcare staff effectively to recognize the events that needed reporting and overcome barriers to reporting adverse events, resulting in a notable increase in the number of incident reports. This demonstrates that the interventions had a substantial impact on improving the reporting rate.

### Interpretation of our findings relative to the literature

The barriers perceived by healthcare staff include the requirement of a considerable amount of information during incident reporting, requirement of writing a review report after incident reporting, and time-consuming reporting process. A technical report published by the WHO regarding incident reporting for ensuring patient safety revealed that the reporting system should be convenient to ensure adequate incident reporting by healthcare professionals^1^. To reduce the burden of reporting on healthcare staff, Evans et al. introduced a reporting system, in which a 3-page reporting format was reduced to a 1-page format^19^. In Taiwan, the data required by the TPR are irreducible; thus, the reporting process is lengthy, which reduces the willingness of staff to report AEs, particularly for near-miss and no-harm events. However, increased reporting speed and reduced reporting time appear to bring about the poor reliability and accuracy of reported information. To resolve this conflict, we selected the TRIZ principle of prior action to develop the interventions. We linked the reporting system with our Healthcare Information System (HIS) and Nursing Information System (NIS), so that the staff only had to enter patients’ medical record numbers and incident dates; the system automatically filled in patient details, such as their basic information and vital signs. These are mandatory data for reporting and were previously a time-consuming part of the process before the intervention, as staff had to search across different systems for information and then manually enter each piece of data. The impact of this intervention can be seen in Table 3, specifically in the reduction of two barriers: “Requirement of a considerable amount of information during incident reporting” and “Time-consuming reporting process.” These decreases highlight the effectiveness of the intervention. Organisations with electronic health record systems potentially have a relatively convenient means of reporting AEs^20^.

The requirement of writing a review report is a reason why healthcare staff members are unwilling to report AEs. However, eliminating this requirement may prevent them from learning from their errors. To resolve this conflict, we selected the TRIZ principle of segmentation; thus, we implemented a policy that only events with SAC scores of 1 to 2 require Root Cause Analysis (RCA) or improvement reports. This markedly reduced the burden on the staff. Routine improvement reports are generally associated with poor analysis and low intervention effectiveness, which result in low levels of system improvement^6^. Thus, the WHO emphasises proper resource allocation is important^1^. The TRIZ principle of segmentation helped us focus on events worth a review.

Our interventions increased the staff’s concerns regarding the negative effects of incident reporting on their colleagues, which ultimately affect teamwork. Effective communication is essential to solve this problem. Teams that openly communicate about errors report incidents more frequently than those that do not communicate openly^21^. Education and training may effectively improve team communication and change the incorrect perception that reporting incidents is a barrier to teamwork^22^. Participation in an additional workshop increased the number of incident reports to 42 times, which was more than the number in the control group^23^. This appears to be an effective approach for administering interventions to groups of departments with the same characteristics; however, conducting such workshops in large healthcare centers is challenging and time consuming because of the sophistication of medical activities, high numbers of departments, and high frequency of interdepartmental cooperation. Our intervention of workshop was developed using the TRIZ principle of mediators. The purposes of this intervention are not only for training but also for developing a long-term partnership with the staff who play the role of mediators. Their tasks in their workplaces are to facilitate incident reporting and to push other patient safety works. In a study from Japan, they appointed as area clinical risk managers in their respective wards or units of responsibility for overseeing the quality of care in their work places, facilitating reporting of incidents, and responding to incidents in their areas by taking corrective actions and communicating as team leaders with patients and family members^24^.

We further investigated whether the recognition of AEs^2,25,26^ and the attitude of supervisors, who lack leadership skills in the context of ensuring patient safety^27^, affect incident reporting. In this regard, two conflict points were identified in our study. One conflict point was that although improving AE recognition enhanced the staff’s sensitivity to reporting AEs, which increased the number of incident reports, it also resulted in extensive nonessential reporting; this increased the burden on the reporting system. To resolve this conflict, we developed a 10- to 15-min-long customised education programme based on the TRIZ principle of segmentation to ensure the accurate reporting of AEs in different work settings. For example, in a teaching programme for surgeons, we related the programme content to actual clinical situations by using the example of patient complaints because of delays in scheduling surgery; this helped surgeons identify the opportunities to improve the system and resolve the problems associated with incident reporting. A study reported that 15-min education programmes are highly effective in conveying the key message in actual clinical settings^26^. The other conflict point was that departmental supervisors were expected to support their staff in reporting AEs; however, some of them lacked leadership skills in the context of ensuring patient safety. We developed a programme based on the TRIZ principle of segmentation, which invited the hospital’s director and deputy directors as educators (mediators) to disseminate information on incident reporting and to assure the staff that the reporting of errors was nonpunitive. This top-down approach enhanced the effectiveness of information dissemination. A Japanese study reported that the participation of a hospital’s director as an educator led to positive outcomes^26^.

In a systematic review of 748 articles, 161 indicated that staff are reluctant to report because they are concerned about the negative consequences of reporting, such as being blamed after reporting; these articles recommended that this concern should be improved as a top priority by organisations^2^. In the present study, the interventions helped our staff overcome the barriers related to the lack of rewards and concerns regarding blame or punishment after incident reporting. The relevant WHO guideline states that the leadership of healthcare organisations must make and commit to policies that establish a safety culture and must ensure the visibility of such commitments^28^. A public commitment by hospital directors may effectively reduce the staff’s concerns regarding incident reporting^26^. However, we believe that considerable changes in current policies as well as new policies are required to make the commitments visible. A major policy may involve rewarding money as an incentive for staff to report AEs. Financial incentives may increase the number of incident reports^29^. The WHO’s World Patient Safety Day Goals 2020–21 highlight the importance of reporting severe AE^30^. In our hospital, incident reporting is voluntary. To increase the reporting of severe AEs, we used the TRIZ principle of segmentation to develop an intervention. The staff received different levels of rewards based on the SAC scores for unintentional errors; higher levels of AE severity indicated higher levels of rewards. The TRIZ principle of segmentation increased the visibility of our commitment and enhanced the flexibility of our reward system to ensure the provision of rewards to the staff members who are truly worthy of incentives. However, an important issue that warrants further discussion is the potential for staff to fabricate adverse events (AEs) in order to receive rewards. To address this concern, we designed a mechanism whereby, after a report is submitted, the system sends a notification to the supervisor of the reporter (in cases of severe medical incidents, notifications are also sent to senior management and legal personnel). The supervisor is responsible for verifying the reported incident.

### Strengths and limitations

This study has several advantages. Incident reporting is an important concern worldwide, but few studies have focused on overcoming barriers of reporting AE and increasing the number of incident reports through various interventions. In particular, very few studies showed the variation on barriers of reporting AE after improvement. To the best of our knowledge, this study is the first to use TRIZ to address patient safety issues and to reveal the effects of TRIZ solutions. Our aim was to establish our TRIZ-based model as a reference for clinical practice and research. However, some limitations of our study must be acknowledged. Because anonymity was prioritised in the preintervention and postintervention surveys to ensure factual reporting, we could not investigate whether the same individuals completed the questionnaires. Furthermore, the interventions overlapped; therefore, we could not separately assess the effects of each intervention.

### Implications for policy, practice, and Research

Future studies may conduct interventions at different time points to separately evaluate the effects of each intervention. Patient perspectives may also be explored as a parameter to evaluate patient safety before and after interventions.

## Conclusions

The implementation of the TRIZ-based model in our healthcare setting significantly enabled staff to overcome barriers to AE reporting and effectively recognize events that needed to be reported, resulting in a notable increase in incident reports. This approach highlights the potential of TRIZ methodology to enhance patient safety practices through creative problem-solving and systematic quality improvements in healthcare. Future research should focus on the long-term sustainability of these interventions and further explore patient perspectives to holistically evaluate the impact on patient safety.

## Electronic supplementary material

Below is the link to the electronic supplementary material.

Supplementary Material 1

## Data Availability

The data underlying this article cannot be shared publicly due to privacy issues. However, they may be provided upon reasonable request. The data can be obtained from the first author, Jiun-Yih Lee (E-mail: m511098001@tmu.edu.tw).

## References

[1] World Health Organization. Patient safety incident reporting and learning systems. *Tech. Rep. Guidance*. https://www.who.int/publications/i/item/9789240010338 (2020).

[2] Archer, S. et al. Development of a theoretical framework of factors affecting patient safety incident reporting: a theoretical review of the literature. *BMJ Open.* **7**, 1–16 (2017). 10.1136/bmjopen-2017-017155 PMC5770969 29284714

[3] Hamed, M. M. M. & Konstantinidis, S. Barriers to Incident Reporting among nurses: a qualitative systematic review. *West. J. Nurs. Res.* **44**, 506–523 (2022). 33729051 10.1177/0193945921999449

[4] Pfeiffer, Y., Manser, T. & Wehner, T. Conceptualising barriers to incident reporting: a psychological framework. *Qual. Saf. Health Care* **19**, (2010). 10.1136/qshc.2008.030445 20558472

[5] Parmelli, E. et al. Interventions to increase clinical incident reporting in health care. *Cochrane Database Syst. Rev.*10.1002/14651858.cd005609.pub2 (2012). 10.1002/14651858.CD005609.pub2 PMC4171121 22895951

[6] Anderson, J. E., Kodate, N., Walters, R. & Dodds, A. Can incident reporting improve safety? Healthcare practitioners’ views of the effectiveness of incident reporting. *Int. J. Qual. Health Care* **25**, 141–150 (2013). 23335058 10.1093/intqhc/mzs081

[7] Berry, L. L., Parasuraman, A., Zeithaml, V. A. & SERVQUAL A multiple-item scale for measuring consumer perceptions of service quality. *J. Retail.* **64**, 12–40 (1988).

[8] Kalaja, R., Myshketa, R. & Scalera, F. Service quality assessment in health care sector: the case of Durres public hospital. *Procedia Soc. Behav. Sci.* **235**, 557–565 (2016).

[9] Hirmukhe, J. Measuring internal customers ’ perception on service quality using SERVQUAL in administrative services. *Int. J. Sci. Res. Publications* **2**, 1–6 (2012).

[10] Su, C. T., Lin, C. & Sen A case study on the application of fuzzy QFD in TRIZ for service quality improvement. *Qual. Quant.* **42**, 563–578 (2008).

[11] LariSemnani, B., Far, M., Shalipoor, R., Mohseni, M. & E. & Using creative problem solving (TRIZ) in improving the quality of hospital services. *Glob J. Health Sci.* **7**, 88–97 (2015). 25560360 10.5539/gjhs.v7n1p88 PMC4796413

[12] Sergei Ikovenko. Training materials for MA TRIZ Level 1. *GEN3 Partners* (2009). https://scholar.google.com/scholar?hl=en&as_sdt=0%2C5&q=Training+materials+for+MA+TRIZ+Level+1&btnG=#d=gs_cit&t=1668129178528&u=%2Fscholar%3Fq%3Dinfo%3AreYKdo-fsxkJ%3Ascholar.google.com%2F%26output%3Dcite%26scirp%3D0%26hl%3Den

[13] Kumar, S. & Bajwa, P. S. Recent trends in Problem solving through TRIZ: a review. *Int. J. Innovative Res. Sci.* **7**, 139–152 (2018).

[14] Lin, S. P., Chen, C. P. & Chen, J. S. Using TRIZ-based method to improve health service quality: A case study on hospital. *2nd International Conference on Economics, Trade and Development IPEDR* 62–66 (2012).

[15] Yener, E. & Altuntas, S. An Approach based on TRIZ methodology and SERVQUAL scale to improve the quality of health-care service: a case study. *Ege Akademik Bakis (Ege Acad. Review)*. **12**, 95–95 (2012).

[16] Kose, I. & Guner, S. A new approach that proposes TRIZ as a creative problem solving technique in health services. *Pressacademia* **7**, 67–79 (2020).

[17] Chiou, C. C., Liu, C. J. & Tsai, J. Integrating PZB model and TRIZ for service innovation of Tele-Healthcare. *Proc. World Acad. Sci. Eng. Technol.* **6**, 577 (2012).

[18] Tonjang, S. & Thawesaengskulthai, N. TRIZ inventive principle in healthcare quality and innovation development. *Int. J. Qual. Reliab. Manage.* ahead-of-print, (2023).

[19] Evans, S. M. et al. Evaluation of an intervention aimed at improving voluntary incident reporting in hospitals. *Qual. Saf. Health Care* **16**, 169–175 (2007). 17545341 10.1136/qshc.2006.019349 PMC2465009

[20] Stovall, M., Hansen, L., van Ryn, M. A. & Critical review moral injury in nurses in the aftermath of a patient safety incident. *J. Nurs. Scholarsh.* **52**, 320–328 (2020). 32222036 10.1111/jnu.12551

[21] Abuosi, A. A. et al. Safety culture and adverse event reporting in Ghanaian healthcare facilities: implications for patient safety. *PLoS One* **17**, 1–18 (2022). 10.1371/journal.pone.0275606 PMC9581362 36260634

[22] Brock, D. et al. Republished: interprofessional education in team communication: working together to improve patient safety. *Postgrad. Med. J.* **89**, 642–651 (2013). 24129031 10.1136/postgradmedj-2012-000952rep

[23] Verbakel, N. J., Langelaan, M., Verheij, T. J. M., Wagner, C. & Zwart, D. L. M. Effects of patient safety culture interventions on incident reporting in general practice: a cluster randomised trial a cluster randomised trial. *Br. J. Gen. Pract.* **65**, e319–e329 (2015). 25918337 10.3399/bjgp15X684853 PMC4408525

[24] Nakajima, K., Kurata, Y. & Takeda, H. A web-based incident reporting system and multidisciplinary collaborative projects for patient safety in a Japanese hospital. *Qual. Saf. Health Care* **14**, 123–129 (2005). 15805458 10.1136/qshc.2003.008607 PMC1743978

[25] Heard, G. C., Sanderson, P. M. & Thomas, R. D. Barriers to adverse event and error reporting in anesthesia. *Anesth. Analg.* **114**, 604–614 (2012). 21821515 10.1213/ANE.0b013e31822649e8

[26] Nakamura, N., Yamashita, Y., Tanihara, S. & Maeda, C. Effectiveness and sustainability of education about incident reporting at a university hospital in Japan. *Healthc. Inf. Res.* **20**, 209–215 (2014). 10.4258/hir.2014.20.3.209 PMC4141135 25152834

[27] Hwang, J., Lee, S. & Park, H. Barriers to the operation of patient safety incident reporting systems in Korean general hospitals. *Healthc. Inf. Res.* **18**, 279–286 (2012). 10.4258/hir.2012.18.4.279 PMC3548158 23346479

[28] World Health Organization. *Global patient safety action Ppan 2021–2030:Towards eliminating avoidable harm E health care* (World Health Organization, 2021).

[29] Feely, J., Moriarty, S. & O’Connor, P. Stimulating reporting of adverse drug reactions by using a fee. *Br. Med. J.* **300**, 22–23 (1990). 2105117 10.1136/bmj.300.6716.22 PMC1661889

[30] World Health Organization. *World patient safety day* 2 (2020).World patient safety day (2020).

