# Supplementary material for: Implementation of a novel TRIZ-based model to increase the reporting of adverse events in the healthcare center
Source: Sci Rep. 2024 Nov 6;14:26905. doi: 10.1038/s41598-024-78661-3 (PMC11542035; doi:10.1038/s41598-024-78661-3)
Supplement: Supplementary file 1 — Supplementary Material 1 [file 41598_2024_78661_MOESM1_ESM.docx]

# PART 1：How much do you know about reporting adverse events (AEs)?

# Test 1：What types of AEs should be reported?

| Question No. | Please Select |
| --- | --- |
| 1. A patient attempted suicide in the hospital but was not injured. Reporting is **not** required. | ☐ Correct ▓ Incorrect |
| 1. A patient fell in the hospital. Reporting is required. | ▓ Correct ☐ Incorrect |
| 1. A patient experienced a sudden change in condition: vomiting and falling to the floor. Reporting is required. | ▓ Correct ☐ Incorrect |
| 1. Upon arrival in the operating room, it was discovered that the surgical site on the patient was incorrectly marked. Reporting is **not** required. | ☐ Correct ▓ Incorrect |
| 1. An incident occurred with the patient during the treatment due to non-compliance with regulations. Reporting is required. | ▓ Correct ☐ Incorrect |
| 1. A patient fell outside the hospital. Reporting is required. | ▓ Correct ☐ Incorrect |
| 1. Blood was drawn from a patient and sent to the laboratory, but the sample was not tested. Reporting is required. | ▓ Correct ☐ Incorrect |
| 1. A fire broke out while using an electric blanket in the hospital. Reporting is required. | ▓ Correct ☐ Incorrect |
| 1. A blood transfusion error occurred (e.g., incorrect blood type, expired blood bag, incorrect amount, or poor quality of blood). Reporting is required. | ▓ Correct ☐ Incorrect |
| 1. Medication was dispensed to a patient (e.g., incorrect dosage, incorrect drug, incorrect measurement, or incorrect administration time), but no harm was caused. Reporting is required. | ▓ Correct ☐ Incorrect |
| 1. A blood transfusion was administered to a patient (e.g., incorrect dosage, incorrect drug, incorrect blood type) when no blood product made contact with the patient's body. Reporting is required. | ▓ Correct ☐ Incorrect |
| 1. A nurse administered the wrong medication (e.g., incorrect dosage, incorrect drug, incorrect measurement, or incorrect method of administration), but it did not involve a patient. Reporting is required. | ▓ Correct ☐ Incorrect |
| 1. A nurse prepared the wrong medication (e.g., incorrect dosage, incorrect drug, incorrect measurement, or incorrect administration method), but it was not given to a patient. Reporting is required. | ▓ Correct ☐ Incorrect |
| 1. A patient committed suicide during hospitalization. Reporting is required. | ▓ Correct ☐ Incorrect |
| 1. The system went down. Reporting is required. | ▓ Correct ☐ Incorrect |
| 1. The elevator was broken. Reporting is required. | ▓ Correct ☐ Incorrect |
| 1. A conflict occurred between a patient or the patient's family and hospital staff. Reporting is required. | ▓ Correct ☐ Incorrect |

# Test 2：：Why incident reporting is crucial for patient safety?

| Question No. | Please Select |
| --- | --- |
| 1. Reporting hospital adverse events can help identify unforeseen incidents or emerging issues. | ▓ Correct ☐ Incorrect |
| 1. Not reporting hospital adverse events prevents timely warnings of new dangerous events and does not raise attention from relevant departments. | ▓ Correct ☐ Incorrect |
| 1. Proactively reporting hospital adverse events can help learn from mistakes and prevent their recurrence. | ▓ Correct ☐ Incorrect |
| 1. To report hospital adverse events and conducting reviews cannot facilitate collaboration among medical teams. | ☐ Correct ▓ Incorrect |
| 1. For a hospital adverse event reporting system to succeed, confidentiality is required to ensure that the reporter's information is not disclosed. | ▓ Correct ☐ Incorrect |

# PART 2：During the process of reporting AEs, have you encountered the following barriers?

| Barriers to reporting AEs | Please Select | Based on your experience or observations of the system, after addressing these barriers, is it possible or has it ever resulted in other issues (the conflict points)? |
| --- | --- | --- |
| 1. Requirement of a considerable amount of information during incident reporting | ☐ YES ☐ NO | **Respond:** |
| 1. Lack of a reward for incident reporting | ☐ YES ☐ NO | **Respond:** |
| 1. Requirement of writing a review report after incident reporting | ☐ YES ☐ NO | **Respond:** |
| 1. Time-consuming reporting process | ☐ YES ☐ NO | **Respond:** |
| 1. Lack of support from supervisors | ☐ YES ☐ NO | **Respond:** |
| 1. Concerns regarding the effects of incident reporting on colleagues and teamwork | ☐ YES ☐ NO | **Respond:** |
| 1. Concerns regarding blame or punishment | ☐ YES ☐ NO | **Respond:** |
